# Protective Effects of Melatonin on *Saccharomyces cerevisiae* under Ethanol Stress

**DOI:** 10.3390/antiox10111735

**Published:** 2021-10-29

**Authors:** Mercè Sunyer-Figueres, Albert Mas, Gemma Beltran, María-Jesús Torija

**Affiliations:** Grup de Biotecnologia Enològica, Departament de Bioquímica i Biotecnologia, Facultat d’Enologia, Universitat Rovira i Virgili, C/Marcel·lí Domingo, 1, 43007 Tarragona, Catalunya, Spain; mercesunyer@gmail.com (M.S.-F.); albert.mas@urv.cat (A.M.); mjesus.torija@urv.cat (M.-J.T.)

**Keywords:** ethanol stress, yeast antioxidant response, melatonin supplementation, ROS accumulation, catalase activity, superoxide dismutase

## Abstract

During alcoholic fermentation, *Saccharomyces cerevisiae* is subjected to several stresses, among which ethanol is of capital importance. Melatonin, a bioactive molecule synthesized by yeast during alcoholic fermentation, has an antioxidant role and is proposed to contribute to counteracting fermentation-associated stresses. The aim of this study was to unravel the protective effect of melatonin on yeast cells subjected to ethanol stress. For that purpose, the effect of ethanol concentrations (6 to 12%) on a wine strain and a lab strain of *S. cerevisiae* was evaluated, monitoring the viability, growth capacity, mortality, and several indicators of oxidative stress over time, such as reactive oxygen species (ROS) accumulation, lipid peroxidation, and the activity of catalase and superoxide dismutase enzymes. In general, ethanol exposure reduced the cell growth of *S. cerevisiae* and increased mortality, ROS accumulation, lipid peroxidation and antioxidant enzyme activity. Melatonin supplementation softened the effect of ethanol, enhancing cell growth and decreasing oxidative damage by lowering ROS accumulation, lipid peroxidation, and antioxidant enzyme activities. However, the effects of melatonin were dependent on strain, melatonin concentration, and growth phase. The results of this study indicate that melatonin has a protective role against mild ethanol stress, mainly by reducing the oxidative stress triggered by this alcohol.

## 1. Introduction

*Saccharomyces cerevisiae* is the main yeast involved in alcoholic fermentation and is widely used not only in industrial fermentation of products such as wine, beer, and bread, but also in the production of bioethanol, a sustainable and clean transportation fuel [1]. During fermentation, yeasts face several stresses, such as osmotic, oxidative, and acidic stresses; nutrient starvation; and the presence of ethanol and other toxic molecules. As these stresses can compromise fermentation performance, an increase in yeast tolerance is a way to enhance the process [2,3]. Therefore, to cope with these fermentation-associated stresses, yeasts have developed specific responses to each stress as well as a general response, the environmental stress response (ESR). These responses are coordinated, and thus the mechanism triggered by one stress can induce a protective response against others, causing a phenomenon called cross-protection [3].

Among all fermentation-associated stresses, ethanol is of capital importance, as its presence is unavoidable in the medium and can lead to a reduction in cell viability, resulting in sluggish or even stuck fermentation [2,4]. Therefore, the effects of this stress and the mechanisms to overcome it have been widely studied [1,5,6]. Due to its structure, ethanol is soluble in both aqueous and lipid phases. For this reason, it can penetrate inside cells but can also be incorporated into cell membranes, increasing membrane fluidity and permeability [7,8]. The accumulation of ethanol and its metabolite acetaldehyde in the cell inhibits its growth due to the inhibition of cell division and the intracellular acidification and denaturation of proteins and enzymes, thereby resulting in altered metabolism [1,9,10,11]. Moreover, ethanol causes an oxidative burst, mainly produced by ROS generation in the mitochondria (H_2_O_2_ and O_2_^•−^) [12,13,14,15], which can induce lipid peroxidation, DNA damage, and oxidative stress [12,16,17]. Therefore, upon ethanol stress, the response to oxidative stress is activated. However, the cellular response to ethanol stress is a complex mechanism mediated by gene expression reprogramming, involving a coordinated action of the ESR, the specific responses to oxidative stress and heat shock [18], and some specific responses to ethanol stress [11], mostly activated by mitochondrial dysfunction [14,19]. 

The oxidative stress response is based on different defense mechanisms that try to maintain cellular ROS concentrations at a basal level. These mechanisms are grouped into enzymatic and nonenzymatic systems [20]. The glutathione system encompasses both enzymatic and nonenzymatic mechanisms, playing a pivotal role in the yeast antioxidant response, although Costa et al. [21] reported that this system is not needed to acquire tolerance to ethanol stress. The main enzymatic defenses against oxidative stress include catalase and superoxide dismutase (SOD). Yeast cells have two catalases (catalase A, encoded by *CTA*, and catalase T, encoded by *CTT*) that decompose H_2_O_2_ to water in the peroxisome and in the cytosol, respectively, and two SODs (Cu/ZnSOD, encoded by *SOD1*, and Mn/ZnSOD, encoded by *SOD2*) that catalyze the conversion of superoxide anion to oxygen and H_2_O_2_ in the cytoplasm and in the mitochondria, respectively [20]. Those enzymes are induced by ethanol stress, as they are pivotal for ethanol tolerance, because they are responsible for eliminating the ROS generated by ethanol [22,23]. Generally, the importance of cytoplasmic SOD in the antioxidant response is much higher than that of the mitochondrial isoforms; however, one exception is ethanol-induced stress [19]. In this condition, although both SODs are activated, the mitochondrial isoform seems to be more necessary to face ethanol stress, probably related to the fact that during ethanol stress, mitochondrial ROS are the primary source of damage [12,14,19,24].

Melatonin (N-acetyl-5-methoxytriptamine) is a bioactive molecule present in most living organisms [25] with widely studied beneficial properties in humans [26,27]. Among the numerous physiological functions of melatonin in humans, several are related to the prevention of some of the disorders related to high ethanol consumption, such as the regulation of circadian rhythms; analgesic, anti-inflammatory, or antistress properties; and modulation of immune functions [28,29,30]. Melatonin also protects human cells, tissues, and organs from ethanol stress, mainly by its antioxidant properties (ROS scavenger action and the activation of the endogen defense system) [29]. Melatonin synthesis by yeast during alcohol fermentation has been reported in several studies [31,32,33,34,35,36,37]. However, little information is available on the synthetic route in yeasts. The first studies reported a route similar to that described for vertebrates [38], but a recent study proposed a putative biosynthetic pathway including some steps described in plants, such as the synthesis of serotonin from tryptophan through tryptamine instead of 5-hydroxytryptophan [39].

A topic under research is whether melatonin confers some advantage to yeast cells during fermentation. Melatonin is reported to act as an antioxidant in *Saccharomyces cerevisiae* [40,41] and non-*Saccharomyces* [42] yeasts and to protect yeast cells against UV radiation [43]. Moreover, recent studies have discovered that melatonin is involved in multiple biological processes in yeasts [41] and could have a signaling role in fermentative metabolism [34,44,45,46]. In the response against oxidative stress, melatonin acts as a direct antioxidant by scavenging ROS and as an indirect antioxidant by decreasing oxidized glutathione and activating genes involved in the oxidative stress response, such as catalase, SOD, glutathione/glutaredoxin, and thioredoxin, leading to reduced lipid peroxidation and higher tolerance to H_2_O_2_ [40,42]. Moreover, the accumulation of some compounds in the media, such as polyphenols or amino acids, has been related to increased yeast tolerance to ethanol [47,48,49,50,51,52]. Resveratrol has been the most studied polyphenol, showing that this compound is able to increase yeast tolerance to ethanol by decreasing lipid peroxidation and SOD activity and by regulating the membrane composition [49].

The production of melatonin by yeast during alcoholic fermentation, together with its protective effect against oxidative stress, suggests the possibility that yeast produces this molecule to protect against fermentation-associated stresses. In fact, the protection against ethanol stress relies partly on antioxidant mechanisms [53], and melatonin confers protection against oxidative stress in human and yeast cells and ethanol stress in human cells [29]. Therefore, it seems interesting to evaluate whether melatonin has a protective effect against ethanol stress in yeast cells. Therefore, the aim of this study was to assess first the effect of different ethanol concentrations on yeast cells and second the effect of melatonin in protecting *S. cerevisiae* cells subjected to ethanol stress. For that, we evaluated mortality, cell recovery, ROS accumulation, lipid peroxidation, and catalase and SOD activities in cells exposed to ethanol stress for different times in the presence and absence of melatonin.

## 2. Materials and Methods

### 2.1. Yeast Strains and Experimental Conditions 

In this study, two strains of *S. cerevisiae* were used: the commercial wine strain QA23 (Lallemand, Montreal, QA, Canada) and the lab strain BY4743 (EUROSCARF collection, Frankfurt, Germany). Yeasts were precultured in yeast extract peptone dextrose (YPD) broth (2% (*w/v*) glucose, 2% (*w/v*) peptone, and 1% (*w/v*) yeast extract (Panreac, Barcelona, Spain)) by incubation at 28 °C with orbital shaking (120 rpm) for 24 h. Then, yeasts were inoculated at an initial optical density measured at a wavelength of 600 nm (OD_600 nm_) of 0.05 in fresh YPD broth with or without melatonin supplementation (0, 5, 25, 50 μM) [TLC grade, purity ≥ 98%, Sigma-Aldrich (St Louis, MO, USA)] and grown until the cells reached the initial exponential phase (OD_600 nm_ 0.5–0.6). These cultures, with or without melatonin supplementation, were submitted to different ethanol concentrations using absolute ethanol (AnalaR NORMAPUR, VWR Chemicals, Radnor, PA, USA). The effect of ethanol on several growth and stress parameters was determined by comparing stressed and unstressed cells in the same growth phase (lag, early exponential, mid-exponential, early stationary, or stationary). Therefore, samples were taken at different growth phases depending on the experiment (specified in the Results section). For the assays of lipid peroxidation and catalase and SOD activities, 10^8^ cells were harvested by centrifugation at 4700 rpm for 5 min at 4 °C, and the pellets were washed with Milli-Q water (Millipore Q-PODTM Advantage A10), centrifuged at 16,000 rpm for 5 min at 4 °C, fast-frozen with liquid nitrogen, and stored at −80 ºC until use. Three biological replicates were employed in all assays, and a nonstressed control without melatonin was performed for each assay.

### 2.2. Determination of Yeast Growth

For both strains, once the cultures with or without melatonin supplementation reached the initial exponential phase (OD_600 nm_ 0.5–0.6), cells were immediately reinoculated in fresh YPD in the presence of different ethanol concentrations (6, 8, 10, 12, 14% (*v/v*)) at OD_600 nm_ 0.2. Yeast growth was monitored for 96 h by measuring OD_600 nm_ every 30 min in a SpectroStar NANO microplate reader (Bmb Labtech, Ortenberg, Germany). For each biological triplicate, five technical replicates were analyzed. From the obtained growth curves, different parameters were evaluated: OD max, growth rate, and the area under the OD-time curve (AUC), or growth potential [54,55]. The growth rate was calculated with the following formula: rate = (log(OD_t_) − log(OD_0_))/t − t_0_, with OD_t_ and OD_0_ being the OD_600 nm_ at specific time and at time 0 h, respectively, and the estimate of AUC was calculated as a metric of the OD distribution as a function of time t.

Additionally, the growth recovery of the cells after being stressed with different ethanol concentrations (8, 10% (*v/v*)) and exposure times was evaluated by inoculating those cells in fresh YPD medium at OD_600 nm_ 0.05. Yeast growth was monitored every 30 min using a SpectroStar NANO microplate reader. For each triplicate, five technical replicates were analyzed. From the obtained growth curves, the different parameters explained above were calculated.

### 2.3. Determination of Mortality Rate

Cell mortality was monitored using propidium iodide (PI) fluorescent staining dye (Invitrogen, Waltham, MA, USA), as specified in the manufacturer’s instructions, with some modifications. Briefly, aliquots of 1 mL of culture were mixed with 1 µg of PI and incubated in darkness at room temperature for 10 min. Then, cells were washed twice with phosphate-buffered saline (PBS, pH 7.4), and fluorescence intensity was measured with the flow cytometer CyFlowspace (Partec, Norderstedt, Germany). Data were acquired with FloMax software (Quantum Analysis GmbH, Münster, Germany) and processed to calculate the percentage of dead cells with WinMDI 2.9 software (Joseph Trotter, Salk Institute for Biological Studies, La Jolla, CA, USA). 

### 2.4. Quantification of ROS 

Reactive oxygen species (ROS) were determined using the fluorescent probe dihydrorhodamine 123 (DHR123), as described in Vázquez et al. [40]. Briefly, samples (0.5 mL) were stained with DHR 123 (Sigma-Aldrich, St Louis, MO, USA) at a final concentration of 10 µg/mL in darkness for 20 min at 120 rpm and 28 °C. Then, the cells were harvested and washed twice with PBS (pH 7.4); ROS were immediately quantified by measuring the fluorescence intensity (geometric mean, Gmean) with a CyFlowspace flow cytometer. Data were acquired with FloMax software, and the median fluorescence intensity was quantified with WinMDI 2.9 software. The mean fluorescence index (MFI) was calculated according to Boettiger et al. [56]: [(Gmean condition) − (Gmean control)]/(Gmean control).

### 2.5. Lipid Peroxidation

The degree of lipid peroxidation was evaluated by the colorimetric determination of thiobarbituric-acid-reactive substances (TBARS) described in Buege and Aust [57], with some modifications. Briefly, cell pellets were resuspended in 450 µL of TCA (trichloroacetic acid 10% (*v/v*)) in PBS and broken using glass beads with five cycles alternating shaking and cooling (30/30 s) using an MBB-16 Mini-Beadbeater (BioSpec Products, Inc., Bartlesville, OK, USA). Then, the cells were incubated for 15 min on ice and centrifuged at 2200 g for 15 min at 4 °C. After this, the protocol of Vázquez et al. [42] was followed: 200 μL of the supernatant was mixed with 200 μL of 2-thiobarbituric acid (TBA, 6.7 g/L) (Sigma-Aldrich) and incubated at 95 °C for 10 min. After cooling at room temperature, the absorbance was measured at 532 nm using the microplate reader SpectroStar NANO. The concentration of TBARS was estimated by referring to a standard curve of 1,1,3,3-tetramethoxylpropane (Sigma-Aldrich), and the results are expressed as nmol of TBARS per mg of protein. 

### 2.6. Antioxidant Enzyme Activities

Protein extracts were obtained following the cell disruption protocol described in Vázquez et al. [42], with minor alterations. Cell pellets were resuspended in 0.5 mL of precooled PBS 50 mM (pH 7) containing one tablet of protease inhibitor cocktail per 10 mL of extraction solution (cOmplet^TM^; Roche, Mannheim, Germany) and disrupted by alternating five cycles of shaking and cooling (30/30 s) using an MBB-16 Mini-Beadbeater in presence of glass beads. Then, homogenates were centrifuged at 14,000 rpm for 5 min at 4 °C, and the supernatant was used to immediately perform the assays in triplicate. 

Total protein content was estimated according to the Bradford method [58]. Briefly, 10 μL of the protein extract was incubated for 15 min with 240 μL of Bradford reagent (Sigma-Aldrich), and absorbance at 595 nm was determined using a SpectroStar NANO microplate reader. Protein content was calculated using a standard curve constructed with bovine serum albumin (BSA; Sigma-Aldrich).

Catalase activity was determined by measuring the decomposition of H_2_O_2_ after 10 min in the presence of the protein extract, according to Góth [59], modified by Hadwan and Abed [60] and with some further modifications. Briefly, 15 μL of protein extract was exposed to 40 μL of H_2_O_2_ (16 mM; Sigma-Aldrich); after incubation for 10 min at 37 °C, 200 μL of ammonium heptamolybdate (32,4 mM; Millipore, Burlington, MA, USA) was added, and the absorbance at 374 nm was measured using a SpectroStar NANO microplate reader. A standard curve was generated with different H_2_O_2_ concentrations (range of 0.5–16 mM) in PBS, and a negative control was assayed for each cell extract.

Superoxide dismutase (SOD) activity was measured by inhibiting the tetrazolium salt WST-1 (2-(4-iodophenyl)-3-(4-nitrophenyl)-5-(2,4-disulfophenyl)-2H-tetrazolium, monosodium salt) reduction by O_2_^•−^ generated by the xanthine/xanthine oxidase system using a commercial assay kit (SOD assay kit, Sigma-Aldrich), as specified by the provider. Briefly, cell extracts diluted 20-fold were mixed with a solution containing WST-1. Then, the xanthine oxidase enzyme solution was added, and the mix was immediately incubated at 37 °C for 20 min, monitoring the increase in absorbance at 450 nm every 1.5 s, using a SpectroStar NANO microplate reader. SOD activity of protein extracts was estimated using a standard curve prepared with known amounts of bovine SOD (range 0–5 U/mL) (Sigma-Aldrich), and the results were expressed as units of SOD per mg protein.

### 2.7. Data Analysis 

Results are presented in figures and tables indicating mean and standard deviations (SD). Three biological replicates were used in all experiments. Data obtained from all the assays were subjected to statistical analysis with a one-way analysis of variance (ANOVA) followed by a Tukey’s post hoc test using GraphPad Prism 7 (GraphPad Software, San Diego, CA, USA). The means of three or more groups were compared in the presence of one independent variable (concentration of ethanol or melatonin). The results were considered statistically significant at a *p*-value < 0.05, and significances are indicated with asterisks (* for *p*-value < 0.05, ** for *p*-value < 0.01, *** for *p*-value < 0.001, and **** for *p*-value < 0.0001) in the supplementary tables. 

## 3. Results

### 3.1. Effect of Different Ethanol Concentrations on S. cerevisiae Growth

**Figure 1:**
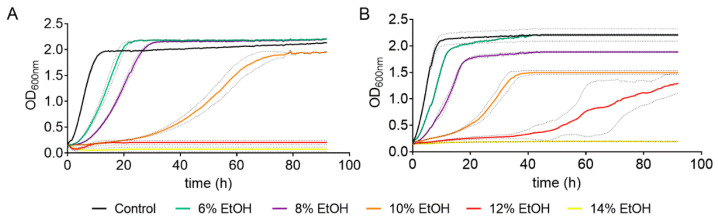
Effect of different ethanol concentrations on the cell growth of *S. cerevisiae* strains BY4743 (**A**) and QA23 (**B**). Ethanol concentrations: 0% (black), 6% (green), 8% (purple), 10% (orange), 12% (red), and 14% (yellow). Standard deviations were indicated with dotted lines.

The effect of ethanol concentration on yeast growth was determined in two *S. cerevisiae* strains: a commercial wine strain (QA23) and a lab strain (BY4743). To do so, both strains were cultivated in YPD medium in the presence of different ethanol concentrations (from 6% to 14%), using a medium without ethanol as a control. The presence of ethanol resulted in a prolonged lag phase and a decrease in the growth rate in both strains (Figure 1 and Figure S1, Table S1). 

As expected, the higher the ethanol concentration was, the longer the yeast growth delay. Indeed, there was a direct correlation between ethanol concentration (from 6% to 10%) and growth rate for both strains (Table S1, Figure S1). However, high ethanol concentrations (10–14%) had a greater effect on the lab strain than on the wine strain (Table S1), and the ethanol concentration that totally suppressed the growth was 12% for the lab strain and 14% for the wine strain. Moreover, the ethanol concentration also affected the OD max obtained. In the case of QA23, the maximum yeast growth decreased almost linearly with the increase in ethanol content, from 6% to 10% ethanol (Figure S1). Surprisingly, in BY4743, low ethanol concentrations (6% and 8%) resulted in higher values of OD max (Figure 1, Table S1), and the decrease was observed only at 10%, as growth was suppressed at 12% and 14%. 

After this first assay, three ethanol concentrations (8%, 10%, and 12%) were chosen to evaluate the effect of ethanol stress in *S. cerevisiae* strains, monitoring different parameters such as cell mortality, cell recovery after stress, and ROS accumulation.

### 3.2. Effect of Ethanol Concentration on Cell Mortality and Growth Recovery

**Figure 2:**
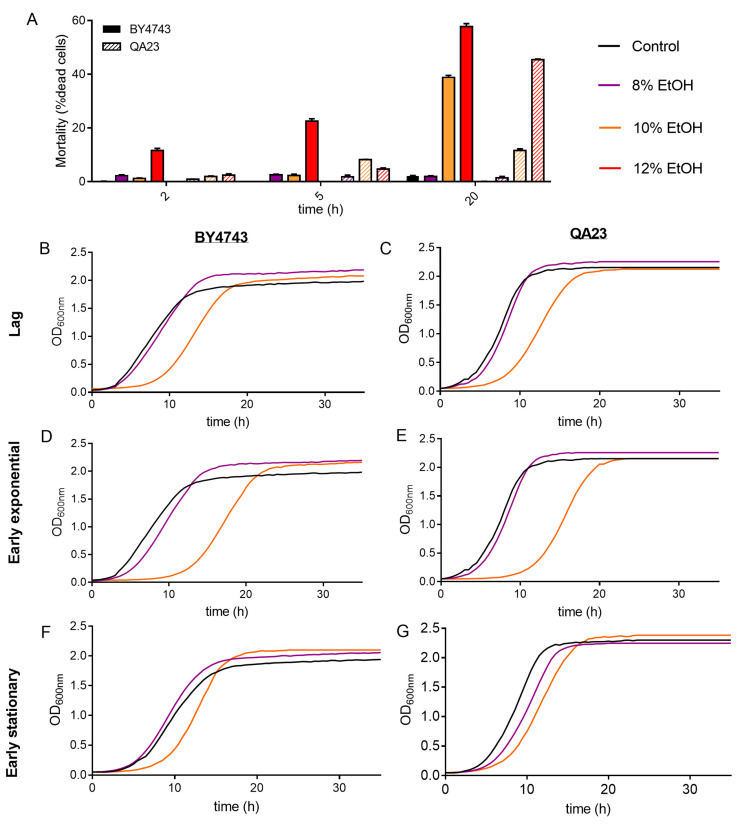
Effect of different ethanol concentrations (0% (black), 8% (purple), 10% (orange)) on the *S. cerevisiae* strains BY4743 (**A**,**B**,**D**,**F**) and QA23 (**A**,**C**,**E**,**G**). (**A**) Mortality rate expressed as the percentage of dead cells (solid columns, BY4743; stripped columns, QA23). (**B**–**G**) growth of cells previously exposed to ethanol and recovered at different growth phases: lag phase (**B**,**C**); early exponential phase (**D**,**E**); early stationary phase (**F**,**G**). Error bars represent standard deviation. The statistical analysis of Graph A is included in Supplementary Table S3.

Cell mortality of the cultures was evaluated on cells exposed to ethanol stress at different concentrations and times. As expected, a higher ethanol content in the medium resulted in an increase in dead cells for both strains, being higher in BY4743 (Figure 2A). As an example, an ethanol concentration of 10% for 20 h resulted in 11.7% of dead cells in QA23 and 38.9% in BY4743. Interestingly, this last percentage of dead cells was similar to that obtained for QA23 with 12% ethanol, indicating a higher tolerance to ethanol stress for the wine strain. 

After that, the growth recovery of the cells after being stressed with different ethanol concentrations and exposure times was evaluated by inoculating those cells in fresh YPD. As the growth curves in the presence of ethanol were delayed in relation to the control (Figure 1), cells were recovered and reinoculated into fresh media according to their growth phase (lag, early exponential, and early stationary phase). 

The recovery of the growth of the stressed cells was affected by the intensity of the stress (ethanol concentration) and by the exposure time to this stress (Figure 2B–G and Figure S2, Table S2). 

The higher the ethanol concentration was, the more time yeast cells needed to recover normal growth, resulting in a longer lag phase, mainly after exposure to 10% and 12% ethanol (Figure 2, Table S2). Indeed, after 8% ethanol exposure, yeast cells grew similarly to nonstressed cells, showing only a slight growth delay under some conditions (in cells recovered at early exponential phase for BY4743 or at early stationary phase for QA23). Our results also show that the growth phase reached by those stressed cells clearly affected growth recovery (Figure S2). After being exposed to 10% ethanol, cell growth recovery was more delayed when cells came from the lag phase and early exponential phase and less affected when cells came from the stationary phase (Figure 2, Table S2). 

### 3.3. Effect of Ethanol on Oxidative Stress Response

**Figure 3:**
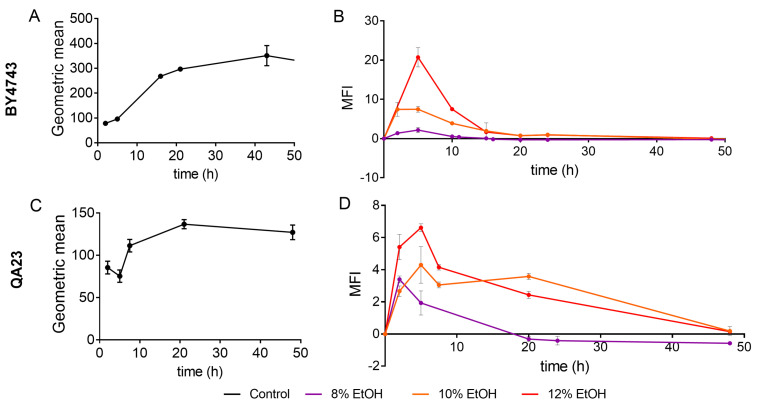
Effect of different ethanol concentrations (0% (black), 8% (purple), 10% (orange), 12% (red)) on ROS (reactive oxygen species) accumulation over time in the BY4743 (**A**,**B**) and QA23 (**C**,**D**) strains. (**A**,**C**) ROS accumulation in the nonstressed cells, expressed as the geometric mean (Gmean). (**B**,**D**) ROS accumulation in stressed cells normalized to nonstressed cells and expressed as Mean Fluorescence Intensity (MFI), [(Gmean stressed cells) − (Gmean control)]/(Gmean control). Error bars represent standard deviation. The statistical analysis of the data is included in Supplementary Table S3.

ROS production in the presence of different ethanol concentrations was evaluated by flow cytometry in both *S. cerevisiae* strains using nonstressed cells as controls. The fluorescence values (Gmean) in nonstressed cultures increased in the mid-exponential phase and reached the maximum fluorescence in the late stationary phase (30–40 h) (Figure 3A,C). 

Both strains followed a similar ROS accumulation pattern, although ROS production was clearly higher in the lab strain (Figure 3A). Ethanol treatments exacerbated ROS generation, presenting the highest fluorescence signal after 5 h of stress exposure (Figure 3B,D). The higher the ethanol concentration was, the higher the ROS production, and this production was especially remarkable in the lab strain treated with 12% of ethanol. In fact, under these conditions, BY4743 presented high mortality and practically no growth (Figure 1A and Figure 2A). In both strains, similar ROS amounts were accumulated in stressed and nonstressed cells in the stationary phase, which was more evident in strain BY4743 (Figure 3).

As we observed that 10% and 12% ethanol seriously compromised the growth and functionality of the lab strain, we analyzed the following oxidative stress indicators (lipid peroxidation, catalase and SOD activities) with a concentration of 8% ethanol in both strains. 

**Figure 4:**
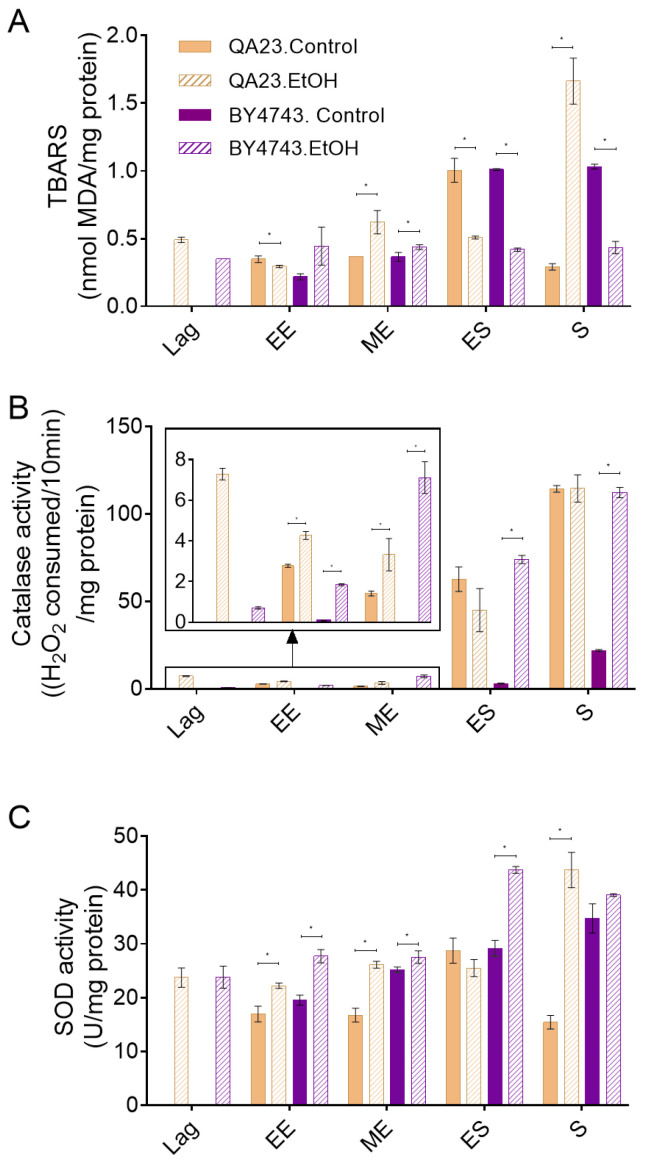
Effect of 8% ethanol on the *S. cerevisiae* strains BY4743 (purple) and QA23 (orange) on (**A**) lipid peroxidation (nmol TBARS/mg protein), (**B**) catalase activity ((H_2_O_2_ consumed/10 min)/mg protein), and (**C**) superoxide dismutase (SOD) activity (U/mg protein). The parameters were calculated at different growth phases after the stress exposure: lag, early exponential (EE), mid-exponential (MD), early stationary (ES), and stationary (S). Solid columns, nonstressed cells; stripped columns, stressed cells. Lag phase was not observed for nonstressed cells. Error bars represent standard deviation, * indicates significant differences between stressed and nonstressed conditions (*p*-value < 0.05), and in Supplementary Table S4, there is a more complete statistical analysis. TBARS stands for thiobarbituric-acid-reactive substances.

Both strains presented a similar profile of lipid peroxidation, with similar levels of TBA reactive substances, in nonstressed cells (Figure 4A). Lipid peroxidation increased with the entry of yeast cells into the stationary phase due to the increase in oxidative stress in this phase. The only difference between strains was that these levels decreased when the stationary phase progressed in the wine strain but were maintained in the lab strain (Figure 4A). Cells exposed to ethanol stress presented a lipid peroxidation profile similar to that of control cells until the early exponential phase and slightly higher levels in the mid-exponential phase. However, in contrast to control cells, practically no changes were observed in TBARS levels due to entrance in stationary phase in any of the strains in the presence of ethanol. Moreover, surprisingly, the QA23 strain sharply increased these levels in the stationary phase, while no changes were detected in BY4743 (Figure 4A). 

As ethanol stress induces ROS accumulation (Figure 3), catalase and SOD activities were measured in the presence and absence of 8% ethanol (Figure 4B,C) to evaluate the effect of ethanol on key enzymes for cell antioxidant defense. Our results show that the catalase activity of nonstressed cells increased when cells entered the stationary phase, in concordance with the other studied parameters (ROS concentration, lipid peroxidation) and with the increase in oxidative stress due to alcoholic fermentation. Although a similar pattern was observed in both strains, the catalase activity in the wine strain was approximately twenty times higher than that in the lab strain. When cells were exposed to ethanol, the general profile of catalase activation was not modified in relation to nonstressed cells and was highly activated during the stationary phase, although some considerations could be made. In the case of QA23, exposure to ethanol induced catalase activity during the lag phase, remaining higher than in nonstressed cells during the exponential phase but achieving a similar final activity at the stationary phase (Figure 4B). On the other hand, in BY4743, the catalase activity in cells exposed to ethanol was clearly higher than that in nonstressed cells throughout the entire process, finishing with similar activity levels to those detected for QA23, both in stressed and nonstressed cells, but five times higher than the activity of nonstressed BY4743 cells (Figure 4B). 

In nonstressed cultures of strain BY4743, SOD activity increased over time, following a similar pattern to catalase activity, although the initial levels were clearly higher; therefore, the increase due to entrance into the stationary phase was less important, just 1.2-fold (Figure 4B,C). In QA23, SOD activity remained mainly unchanged during the exponential phase, increasing only in the early stationary phase. As in BY4743, QA23 presented high levels of SOD activity from the beginning of the growth; thus, the entrance to stationary phase provoked only a 1.7-fold activity induction. In both strains, ethanol exposure did not much change the activity profile of SOD, being maximal in the stationary phase, although with higher values than in the control condition. Moreover, a displacement of this maximum activity was observed, which was in the early stationary phase for BY4743 and in the stationary phase for QA23 (Figure 4C). 

Although exposure to 8% ethanol was not lethal for the studied strains, it clearly affected their cell growth and mortality, their cell oxidative state, and the activity of some enzymes associated with the antioxidant response. The presence of melatonin has previously been described to have an antioxidant role in yeast cells [40,42]. For these reasons, we evaluated the effect of melatonin in yeast cells exposed to ethanol stress at the lag phase, early exponential phase, and early stationary phase (Figure S3). 

### 3.4. Effect of Melatonin on Cell Viability under Ethanol Stress

To evaluate the effect of melatonin on the viability of yeasts exposed to ethanol stress, *S. cerevisiae* cultures were grown in the presence of different melatonin concentrations (5, 25, 50 μM). When the cultures reached the early exponential phase, cells were stressed with 8% of ethanol to analyze cell recovery and mortality after different incubation times in the presence of ethanol. Moreover, cultures grown in the presence of melatonin were transferred to fresh media containing 8% ethanol, and cell growth was monitored. 

**Figure 5:**
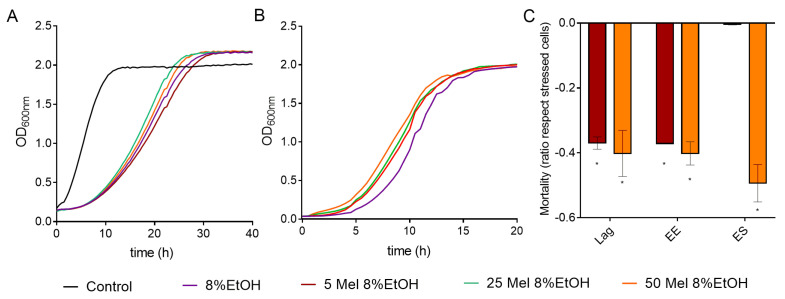
Effect of melatonin (Mel) supplementation of BY4743 cells exposed to 8% ethanol on (**A**) cell growth, (**B**) growth of cells previously exposed to ethanol and recovered at exponential phase, and (**C**) mortality of cells exposed to ethanol in lag, early exponential (EE), and early stationary (ES) phases (ratio of mortality in stressed cells with Mel vs. stressed cells without Mel). Nonstressed cells (black) and stressed cells with Mel supplementation: 0 (purple), 5 (maroon), 25 (green) or 50 (orange) µM. Error bars represent standard deviation, and * represents significant differences between stressed cells with and without melatonin. In Supplementary Table S6 there is a more complete statistical analysis of Graph C.

Melatonin had a slight effect on the growth of cells exposed to ethanol by increasing (50, 25 µM) or decreasing (5 µM) the area under the curve (AUC), although these changes were not significant (Figure 5A and Figure S4A, Table S5). Melatonin pretreatment also modified the OD max obtained in the QA23 strain, with a slight increase when cells were grown with 25 and 50 µM melatonin and a decrease with 5 µM melatonin (Figure S4A, Table S5).

We also tested the effect of melatonin on the growth recovery of stressed cells at different exposure times (Figure 5B and Figure S4B, Table S5). In BY4743 cells, all melatonin concentrations significantly shortened the lag phase at the early exponential phase (Figure 5B). For strain QA23, similar growth curves were obtained for stressed cells treated with or without melatonin, although the values of AUC and OD max were slightly increased by melatonin (Figure S4B, Table S5). These results suggest that melatonin could modulate the growth recovery of cells exposed to ethanol stress. 

Low concentrations of melatonin (5 µM) significantly decreased the mortality triggered by 8% ethanol in lag and early exponential phases in BY4743, while high concentrations (50 µM) also decreased it in the early stationary phase (Figure 5C). A similar effect was observed in QA23 (Figure S4C). Therefore, in both strains, the presence of melatonin decreased the cell mortality of cells exposed to ethanol stress, mainly during the initial growth phases.

### 3.5. Effect of Melatonin as an Antioxidant Shield under Ethanol Stress

Once it was shown that co-treatment with melatonin improved the viability of ethanol-treated cells and ethanol-induced oxidative stress, we wanted to determine whether melatonin could protect cells from this oxidative stress caused by the presence of ethanol (8%). Therefore, we studied the effect of melatonin on different parameters related to oxidative stress.

**Figure 6:**
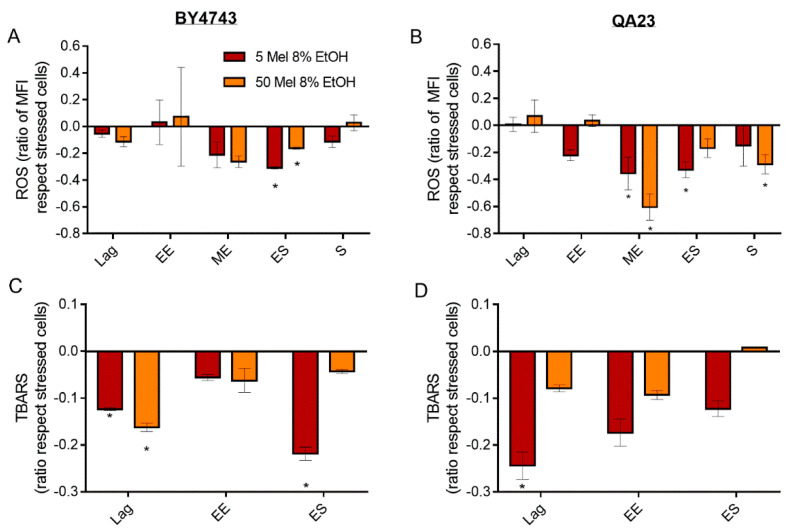
Effect of melatonin (Mel) supplementation (5 (red) or 50 (orange) µM) of BY4743 (**A**,**C**) and QA23 (**B**,**D**) cells exposed to 8% ethanol in lag, early exponential (EE), mid-exponential (ME), early stationary (ES), and stationary (S) phases, on (**A**,**B**) ROS accumulation (ratio of MFI of stressed cells with Mel vs. stressed cells without Mel) and (**C**,**D**) lipid peroxidation (ratio of TBARS of stressed cells with Mel vs. stressed cells without Mel). Error bars represent the standard deviation, * indicates significant differences with respect to stressed cells without melatonin (*p*-value < 0.05), and in Supplementary Table S6, there is a more complete statistical analysis. ROS stands for reactive oxygen species, and TBARS stands for thiobarbituric-acid-reactive substances.

Both melatonin concentrations tested (5 and 50 µM) decreased ROS accumulation during the initial growth of BY4743 in the presence of ethanol, which was significant at entry to the stationary phase (Figure 6A). At this point, stressed cells treated with melatonin presented even lower ROS accumulation than nonstressed cells (Figure 3B). However, as the stationary phase progressed, melatonin-treated cells increased ROS levels until reaching levels similar to those of stressed cells without melatonin (Figure 6A). The profile of ROS generation in QA23 was similar to that in BY4743, with a significant reduction in ROS accumulation during the mid-exponential and early stationary phases at both concentrations of melatonin (Figure 6B). However, these same melatonin concentrations had a lower effect in cells submitted to higher ethanol concentrations (10%), as although a significant ROS reduction was observed in the early exponential phase, the ROS levels increased during the stationary phase (data not shown).

Melatonin, regardless of the concentration, reduced lipid peroxidation provoked by ethanol stress in both strains (Figure 6C,D). This effect was significant in the lag phase and decreased over time, except for BY4743 in the early stationary phase in the presence of 5 μM of melatonin. 

Once it was proven by a decrease in oxidative damage that melatonin has some protective effect on cells stressed with ethanol, we wanted to assess whether melatonin also affected the activity of enzymes related to antioxidant capacity, such as catalase and SOD.

**Figure 7:**
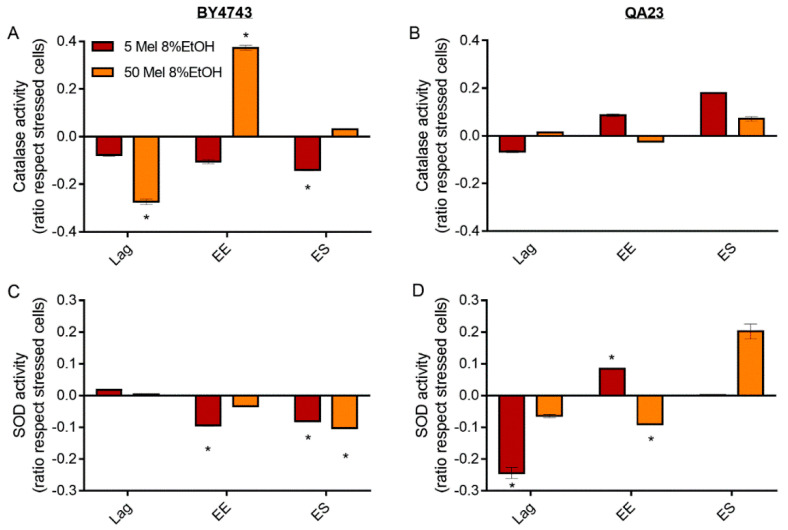
Effect of melatonin (Mel) supplementation (5 (red) or 50 (orange) µM) of BY4743 (**A**,**C**) and QA23 (**B**,**D**) cells exposed to 8% ethanol in lag, early exponential (EE), and early stationary (ES) phases on (**A**,**B**) catalase activity and (**C**,**D**) superoxide dismutase (SOD) activity (ratio of the enzymatic activity of stressed cells with Mel vs. stressed cells without Mel). Error bars represent the standard deviation, * indicates significant differences with respect to stressed cells without melatonin (*p*-value < 0.05), and in Supplementary Table S6, there is a more complete statistical analysis.

In BY4743 cells, the presence of low melatonin concentrations (5 µM) provoked a progressive decrease in catalase activity over time compared to the stressed cells without melatonin, which was significant in the early stationary phase. In contrast, the presence of high melatonin concentrations (50 µM) rapidly decreased catalase activity in the lag phase and increased it during the early exponential phase, while no difference was observed at the early stationary phase (Figure 7A). On the other hand, in QA23, a low melatonin concentration initially decreased catalase activity at the lag phase but increased catalase activity at the early exponential and early stationary phases, during which its activity was higher than that in stressed cells without melatonin (Figure 7B). Similar results were obtained with a lower melatonin concentration in the medium (1 μM), while higher concentrations practically did not modify the catalase activity in this strain (data not shown).

For SOD activity, both melatonin concentrations triggered the same behavior in BY4743 cells; melatonin slightly increased SOD activity at lag phase and decreased it at early exponential and stationary phases (Figure 7C). Interestingly, in the wine strain, melatonin treatment of stressed cells caused an inverse profile. SOD activity decreased at the lag phase (especially significant for low melatonin concentrations), and afterwards, this activity increased until the early stationary phase (Figure 7D).

## 4. Discussion

Melatonin exhibits antioxidant properties in different organisms, including humans and yeast [28,30,40,42]. In yeasts, this molecule has been reported to be produced during fermentation to face the associated stresses [31,32,33,34,40,42]. One of the main stress factors that yeast cells encounter during fermentation is the production and accumulation of ethanol in the medium, which could be toxic to yeast cells. Although *S. cerevisiae* possesses inherent tolerance to ethanol, some toxic effects have been associated with ethanol accumulation, such as an increase in oxidative stress, inactivation of related enzymes, dysfunctional mitochondrial metabolism, and interference on the cellular membrane and wall [1,11,15,16,22,55,61]. Moreover, ethanol accumulation is one the main causes described to explain the death of non-*Saccharomyces* species during wine production [62,63]. Thus, as high ethanol concentrations induce oxidative stress, it seems plausible that melatonin, due to its role as an antioxidant, could protect against this stress. Moreover, this molecule is reported to protect humans from the oxidative stress provoked by ethanol on tissues and organs (reviewed in Kurhaluk and Tkachenko, [29]), so we evaluated whether melatonin also had a protective effect against the oxidative stress provoked by ethanol in yeast cells. For this reason, we first studied the effect of different ethanol concentrations in two *S. cerevisiae* strains (a wine strain and a lab strain, QA23 and BY4743, respectively) on different parameters related to cell viability and oxidative stress and then studied the possible protective effect of two melatonin concentrations (5 and 50 µM) on cells exposed to ethanol. 

As commented previously, ethanol is a metabolite produced by yeast that can become toxic when a threshold is surpassed, and different yeast strains can show very different abilities to grow and survive in the presence of ethanol [5,55,64]. Therefore, the two *S. cerevisiae* strains were first confronted with a range of ethanol concentrations to determine their cellular response and tolerance to this stress. As expected, increasing ethanol concentrations produced stronger effects on yeast viability, cell recovery, and ROS accumulation (also reported in several studies, such as Cheng et al. [48], Martínez-Alcántar et al. [65], and Navarro-Tapia et al. [66]) until reaching the inhibitory concentration, at which point cell growth was totally inhibited. Similar behavior in response to ethanol stress was observed in the two strains, although the wine strain exhibited higher tolerance to high alcohol concentrations than the lab strain. In the case of the lab strain, 12% ethanol totally suppressed its growth, whereas in the wine strain, 14% of ethanol was necessary to totally inhibit it. Additionally, the wine strain presented a lower percentage of dead cells; more QA23 cells endured and thrived in ethanol stress, requiring a higher percentage of ethanol to achieve a similar percentage of dead cells to that in BY4743. These results agree with those of Lairón-Peris et al. [55], who reported that wine yeast strains were among the most ethanol-tolerant *S. cerevisiae* strains. Additionally, the wine strain also generated a lower quantity of ROS, both at entry into the stationary phase and in response to ethanol treatment. These results confirmed that the wine strain was better adapted to withstand ethanol stress and other stresses originating during wine fermentation, as also reported in Pais et al. [67] with other wine and lab strains. 

In general, ethanol-stressed cells exhibited higher mortality and oxidative stress (shown by ROS accumulation, lipid peroxidation, and the activation of some antioxidant enzymes) and lower cell growth and recovery capacity than nonstressed cells. There was only one exception: entry into early stationary phase, in which nonstressed cells increased the markers of oxidative damage even more than stressed cells. Cells in the stationary phase are stressed by the lack of nutrients and by the accumulation of toxic metabolites [68,69], leading to an increase in oxidative stress markers [70,71] and the activation of the ESR [3,18,72], which includes mechanisms for stress resistance, such as *SOD2* [68]. Therefore, this response is triggered in the stationary phase in nonstressed cells but earlier in stressed cells. During alcoholic fermentation, ethanol accumulates over time in the wine environment, and a clear correlation has been established between ethanol and oxidative stress in yeast [13,16,22]. Ethanol stress induced a quick formation of ROS and lipid peroxidation. This ROS generation started just after the application of the stress, reaching its maximum after 5 h of the treatment with a subsequent decline. Other studies also reported an accumulation of H_2_O_2_ and O_2_^•−^ in mitochondria after short ethanol exposure times [12,65,73], and in poorer media, this ROS increase can last longer [13,17]. ROS are toxic to yeast, as they inhibit metabolic processes and prevent cellular growth, so the fact that ROS levels were higher in cells under early exponential growth could be the reason why these cells need more time to recover normal growth. The induction of membrane lipid peroxidation by ethanol stress was also reported in Gharwalova et al. [49], Fierro-Risco et al. [74], and Gupta et al. [75]. Thus, ethanol seemed to accelerate ROS formation, mainly in the mitochondria, causing lipid peroxidation in the membranes that was maintained over time. On the other hand, the rapid decrease in ROS levels after reaching the maximum in the early exponential phase could be explained by the fact that ROS can stimulate mitophagy, leading to the elimination of damaged and dysfunctional mitochondria and thereby contributing to a decrease in H_2_O_2_ and O_2_^•−^ [14]. Thus, this could result in the protection of the cell from ROS damage, a decrease in cell mortality, and an increase in tolerance to ethanol.

Additionally, the response to oxidative damage triggers the synthesis of enzymes that are able to detoxify ROS, such as catalases or SOD, among others [76,77]. Our results indicate that in nonstressed cells, antioxidant enzymes, especially catalase, are mostly activated in the entry to stationary phase, when the sugar present in the medium is low and the oxidative stress is higher. These data are concordant with the studies that describe that antioxidant defenses are mainly repressed by glucose during exponential growth and derepressed when cells approach the diauxic shift to prepare for the increase in oxidative stress [22,78]. On the other hand, the cells treated with ethanol activated the antioxidant machinery earlier, to counteract the effects of oxidative stress at the beginning of growth, thereby exhibiting higher oxidative stress tolerance [78]. The induction of these enzymes by ethanol has been widely reported [12,22,49,74,79], highlighting the importance of both SODs and the cytosolic catalase for ethanol tolerance [13,15,21,23,48,53]. In this study, catalase was clearly activated during the stationary phase, but SOD also presented high activity during the exponential phase, with a profile similar to that of lipid peroxidation, suggesting that SOD is activated before catalase. This is not surprising, because SOD is in charge of the detoxification of the O_2_^•−^ anion by transforming it in H_2_O_2_, which is subsequently detoxified by catalases [12,14]. 

In general, the presence of melatonin lightened the effect of 8% ethanol on cell growth. Cells treated with a range of 5 to 50 µM melatonin exhibited improved cell viability when submitted to 8% ethanol. In cells pretreated with melatonin and exposed to ethanol in early growth stages, cell mortality was decreased, and better cell recovery was obtained. When the cells were exposed to ethanol in the stationary phase, those effects of melatonin were lessened, probably due to an adaptation of the cells and an activation of the ESR, which was reflected in a lower mortality due to ethanol presence. These results seem to point towards an early protective response of melatonin against ethanol stress, similar to the one observed against oxidative stress, which was activated after 45 min [40]. 

Our results also show that cells grown with melatonin and exposed to ethanol stress had less oxidative damage than stressed cells without melatonin, as ROS accumulation and lipid peroxidation were lower. Similar results were previously reported for cells subjected to oxidative stress in the presence and absence of melatonin [40,42] and to ethanol stress in the presence and absence of resveratrol [49]. A higher concentration of melatonin did not always confer higher protection, as was observed for oxidative stress in Vázquez et al. [40].

Melatonin has been found to interfere with cellular antioxidant activities and the transcriptional machinery, more specifically, modulating the genes of the antioxidant response [40,41,43]. These antioxidant activities were generally decreased by melatonin in stressed cells, although in the wine strain, after an initial decrease, the activities started to increase over time. In a previous study, an increase in catalase activity was observed in nonstressed cells treated with melatonin, suggesting a pro-oxidant role of this compound [42]. In this study, similar results were obtained, as melatonin in nonstressed QA23 cells induced a similar pattern to that of ethanol-stressed cells, by increasing catalase activity in the exponential phase and decreasing catalase activity in the stationary phase. These results again suggest a possible pro-oxidant effect of melatonin, which could confer resistance to further oxidative stress exposure [42,43]. Therefore, as those cells were previously grown in melatonin before the stress was applied, the presence of melatonin could have activated the antioxidant response, making them more prepared to endure ethanol stress and reducing the need to activate those enzymes against future stresses. Gharwalova et al. [49] found a similar decrease in SOD activity in ethanol-stressed cells treated with resveratrol, and Estruch et al. [80] found a similar decrease in healthy men after red wine intake, suggesting a reduction in enzyme activity when not necessary to save energy. The induction of SOD in the wine strain by melatonin in the early stationary phase seems to validate this idea. In this strain, the presence of melatonin caused a fast activation of antioxidant machinery after ethanol stress, neutralizing the stress and quickly relaxing the defense system. Therefore, when cells entered stationary phase, they needed to again activate the antioxidant machinery. In contrast, in the lab strain, the response activated by melatonin was less efficient, and antioxidant defenses were still activated when cells entered stationary phase.

In conclusion, ethanol increased the production of ROS and lipid peroxidation and triggered the activation of antioxidant defense, which was consistent with previous studies. However, in cells treated with melatonin, such damage was attenuated by the antioxidant capacity of this molecule, which was able to scavenge ROS, reducing their noxious effects, such as lipid peroxidation, and increasing ethanol tolerance. The mitigation of oxidative stress by the presence of melatonin reduced the activity of some antioxidant enzymes, thereby resulting in a lower oxidative stress response. Therefore, these results suggest that cells grown in the presence of melatonin are better prepared to endure ethanol stress. However, other important targets of ethanol stress, such as lipid membranes, mitochondria, or the accumulation of reserve carbohydrates (trehalose and glycogen) are reported to be affected by melatonin supplementation in yeast cells. Therefore, those targets should be evaluated to fully understand the mechanism by which melatonin confers protection against ethanol stress.

## Data Availability

All the data is available within the article and Supplementary Materials.

## Supplementary Materials

The supplementary materials are available online at www.mdpi.com/article/10.3390/antiox10111735/s1. Figure S1: Linear regressions correlating ethanol concentration (6–10%) with growth rate (circles, h^−1^), maximum OD (squares, OD max) and area under the curve (triangles, AUC) obtained from the growth curves of the QA23 strain. Figure S2: Growth of BY4743 (A,B) and QA23 (C,D) cells previously exposed to ethanol (0%, B, D; 8%, A, C) and recovered at different growth phases: lag phase, early exponential phase, mid-exponential phase, early stationary phase and stationary phase. No lag phase was observed for nonstressed cells. Figure S3: Growth curve of BY4743 (purple) and QA23 (orange) strains with 0% (continuous line) and 8% (discontinuous line) ethanol. Time 0 h represents the moment in which ethanol stress was applied. Figure S4: Effect of melatonin (Mel) supplementation on QA23 cells exposed to 8% ethanol on (A) cell growth, (B) growth of cells previously exposed to ethanol and recovered at exponential phase and (C) mortality of cells exposed to ethanol until lag, early exponential and early stationary phase (ratio of mortality in stressed cells with Mel vs stressed cells without Mel). Nonstressed cells (black) and stressed cells with Mel supplementation: 0 (purple), 5 (maroon), 25 (green) or 50 (orange) µM. Error bars represent standard deviation, and * significant differences between stressed cells with and without melatonin. Table S1: Effect of different ethanol concentrations on the growth of S. cerevisiae QA23 and BY4743. The parameters analyzed were growth rate (h-1), maximum OD (ODmax) and area under the curve (AUC) calculated until 80 h of growth. The linear regression values (slope and r2) were calculated for concentrations in the range of 6–10% ethanol. Mean and standard deviation (SD) values are represented, * indicates significant differences between stressed and nonstressed conditions (* for *p*-value < 0.05; *** for *p*-value < 0.001 and **** for *p*-value < 0.0001). Table S2: Effect of different ethanol concentrations (0%, 8%, 10%) on the growth of BY4743 and QA23 cells previously exposed to ethanol and recovered at different growth phases: lag phase; early and mid-exponential phases; early stationary and stationary phases. The parameters analysed were growth rate (h-1), maximum OD (ODmax) and area under the curve (AUC) calculated until 12 h of growth. Lag phase was not observed for nonstressed cells (indicated with a slash “-“). ND stands for not determined. Mean and standard deviation (SD) values are represented, * indicates significant differences between stressed and nonstressed conditions (* for *p*-value < 0.05; ** for *p*-value < 0.005; and **** for *p*-value < 0.0001). Table S3: Effect of different ethanol concentrations (0%, 8%, 10%, 12%) on mortality and ROS (reactive oxygen species) accumulation over time in the BY4743 and QA23 strains. Mortality rate expressed as the percentage of dead cells, ROS accumulation expressed as the geometric mean (Gmean). Mean and standard deviations are expressed. * indicates significant differences between stressed and nonstressed conditions (* for *p*-value < 0.05; ** for *p*-value < 0.01, *** for *p*-value < 0.001 and **** for *p*-value < 0.0001). ND stands for not determined. Table S4: Effect of 8% ethanol on the S. cerevisiae strains BY4743 and QA23 on lipid peroxidation (nmol TBARS/mg protein), catalase activity ((H2O2 consumed/10 min)/mg protein) and superoxide dismutase (SOD) activity (U/mg protein). The parameters were calculated at different growth phases after the stress exposure: lag, early exponential (EE), mid-exponential (ME), early stationary (ES) and stationary (S). Lag phase was not observed for nonstressed cells. Mean and standard deviations are expressed. * indicates significant differences between stressed and nonstressed conditions (* for *p*-value < 0.05; ** for *p*-value < 0.01, *** for *p*-value < 0.001 and **** for *p*-value < 0.0001). TBARS stands for thiobarbituric acid-reacting substances. Table S5: Effect of melatonin supplementation (0, 5, 25, 50 µM) on the growth curve and recovery of S. cerevisiae strains BY4743 and QA23 after exposure to 8% ethanol. The parameters analysed were growth rate (h^−1^), maximum OD (ODmax) and area under the curve (AUC) until 20 h (growth curve) and 12 h (recovery) of growth. These parameters were calculated at different growth phases after the stress exposure: lag, early exponential (EE), and early stationary (ES). ND: Not determined. Mean and standard deviation (SD) values are represented, * indicates significant differences between stressed and nonstressed conditions (* for *p*-value < 0.05; ** for *p*-value < 0.005; *** for *p*-value < 0.001 and **** for *p*-value < 0.0001). Table S6: Effect of melatonin (Mel) supplementation (5 or 50 µM) on BY4743 and QA23 cells exposed to 8% ethanol until lag, early exponential (EE), mid-exponential (ME), early stationary (ES) and stationary (S) phases, on mortality, ROS (reactive oxygen species) accumulation, lipid peroxidation, catalase and superoxide dismutase (SOD) activity. The parameters are expressed as ratio of the values of stressed cells with Mel vs. stressed cells without Mel. Lag phase was not observed for nonstressed cells. Mean and standard deviations are expressed. * indicates significant differences with respect to stressed cells without melatonin (* for *p*-value < 0.05; ** for *p*-value < 0.01, *** for *p*-value < 0.001 and **** for *p*-value < 0.0001). TBARS stands for thiobarbituric acid-reacting substances.
