# Supplementary material for: Protective Effects of Melatonin on Saccharomyces cerevisiae under Ethanol Stress
Source: Antioxidants (Basel). 2021 Oct 29;10(11):1735. doi: 10.3390/antiox10111735 (PMC8615028; doi:10.3390/antiox10111735)
Supplement: Supplementary file 1 [file antioxidants-10-01735-s001.zip › antioxidants-1434607-supplementary.pdf]

Supplementary Material

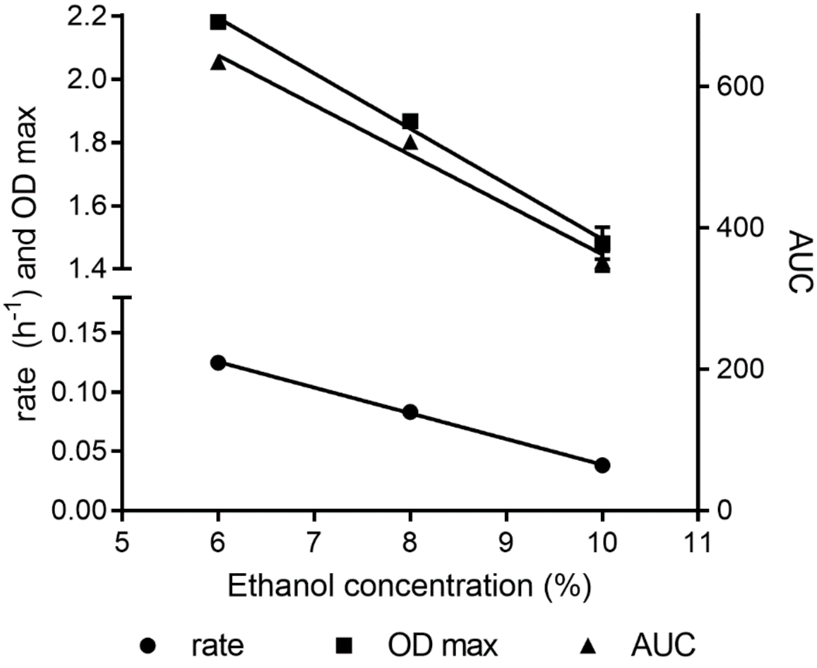

Figure S1: Linear regressions correlating ethanol concentration (6-10%) with growth rate (circles, h^{-1}), maximum OD (squares, OD max) and area under the curve (triangles, AUC) obtained from the growth curves of the QA23 strain.

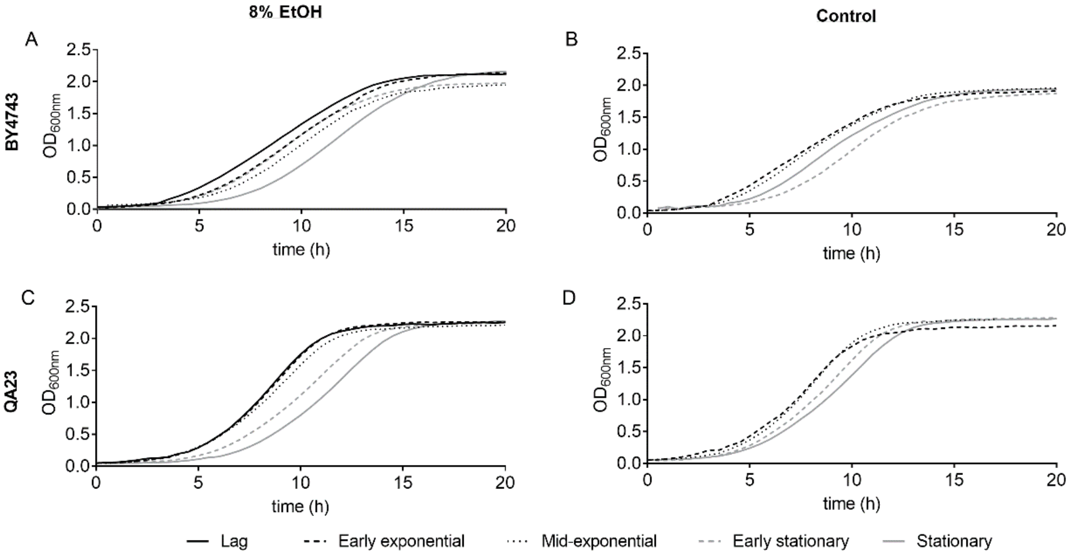

Figure S2: Growth of BY4743 (**A**,**B**) and QA23 (**C**,**D**) cells previously exposed to ethanol (0%, B, D; 8%, A, C) and recovered at different growth phases: lag phase, early exponential phase, mid-exponential phase, early stationary phase and stationary phase. No lag phase was observed for nonstressed cells.

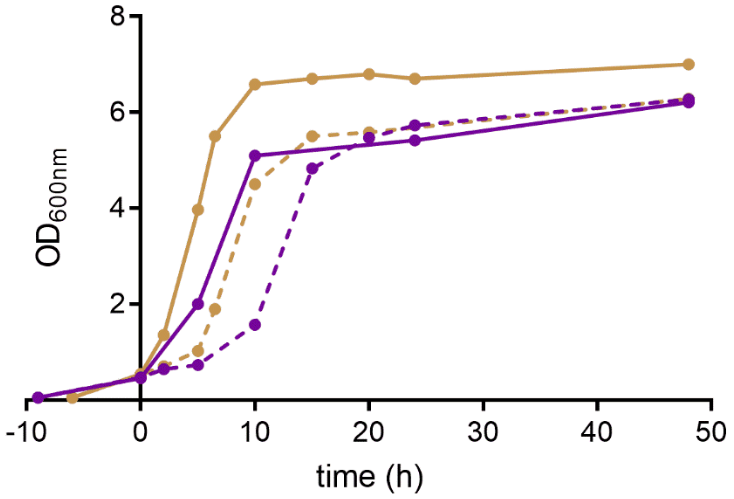

Figure S3: Growth curve of BY4743 (purple) and QA23 (orange) strains with 0% (continuous line) and 8% (discontinuous line) ethanol. Time 0 h represents the moment in which ethanol stress was applied.

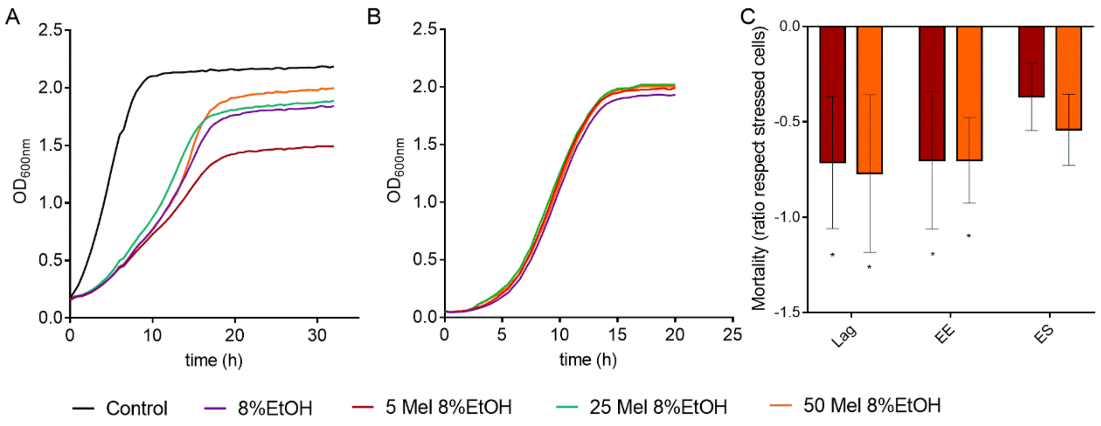

Figure S4: Effect of melatonin (Mel) supplementation on QA23 cells exposed to 8% ethanol on (**A**) cell growth, (**B**) growth of cells previously exposed to ethanol and recovered at exponential phase and (**C**) mortality of cells exposed to ethanol until lag, early exponential and early stationary phase (ratio of mortality in stressed cells with Mel vs stressed cells without Mel). Nonstressed cells (black) and stressed cells with Mel supplementation: 0 (purple), 5 (maroon), 25 (green) or 50 (orange) µM. Error bars represent standard deviation, and * significant differences between stressed cells with and without melatonin.

Table S1: Effect of different ethanol concentrations on the growth of *S. cerevisiae* QA23 and BY4743. The parameters analyzed were growth rate (h^{-1}), maximum OD (ODmax) and area under the curve (AUC) calculated until 80 h of growth. The linear regression values (slope and r^2) were calculated for concentrations in the range of 6-10% ethanol. Mean and standard deviation (SD) values are represented, * indicates significant differences between stressed and nonstressed conditions (* for p-value < 0.05; *** for p-value < 0.001 and **** for p-value < 0.0001).

	**Rate (h-1)**		**ODmax**		**AUC**	
**Ethanol concentrations (%)**	BY4743	QA23	BY4743	QA23	BY4743	QA23
**0**	0.148 ± 0.003	0.173 ± 0.001	2.132 ± 0.004	2.202 ± 0.114	592.626 ± 4.235	689.847 ± 2.563
**6**	0.090 ± 0.003 ****	0.125 ± 0.006 ****	2.204 ± 0.004 *	2.181 ± 0.006	579.542 ± 6.733	634.705 ± 2.065
**8**	0.065 ± 0.001 ****	0.083 ± 0.006 ****	2.200 ± 0.010*	1.868 ± 0.020 ****	532.495 ± 4.636 ***	521.683 ± 1.5223 ****
**10**	0.025 ± 0.002 ****	0.038 ± 0.004 ****	1.955 ± 0.016 ****	1.481 ± 0.050 ****	261.923 ± 21.719 ****	353.108 ±14.249 ****
**12**	0.005 ± 0.001 ****	0.035 ± 0.001 ****	0.464 ± 0.043 ****	1.413 ± 0.157 ****	141.599 ± 12.270 ****	155.192 ± 47.214 ****
**14**	0.004 ± 0.001 ****	0.015 ± 0.003 ****	0.423 ± 0.058 ****	0.201 ± 0.004 ****	104.676 ± 12.760 ****	60.694 ± 1.174 ****
**LINEAR REGRESSION VALUES**						
**slope**	-0.016	-0.022	-0.062	-0.175	-79.405	-70.399
**r2**	0.983	0.999	0.760	0.996	0.858	0.987

Table S2: Effect of different ethanol concentrations (0%, 8%, 10%) on the growth of BY4743 and QA23 cells previously exposed to ethanol and recovered at different growth phases: lag phase; early and mid-exponential phases; early stationary and stationary phases. The parameters analysed were growth rate (h^{-1}), maximum OD (ODmax) and area under the curve (AUC) calculated until 12 h of growth. Lag phase was not observed for nonstressed cells (indicated with a slash "-"). ND stands for not determined. Mean and standard deviation (SD) values are represented, * indicates significant differences between stressed and nonstressed conditions (* for p-value < 0.05; ** for p-value < 0.005; and **** for p-value < 0.0001).

	**Control**			**8% ethanol**			**10% ethanol**		
**time (h)**	**Rate (h^{-1})**	**OD_{max}**	**AUC**	**Rate (h^{-1})**	**OD_{max}**	**AUC**	**Rate (h^{-1})**	**OD_{max}**	**AUC**
	**BY4743**								
**Lag**	-	-	-	0.202 ± 0.000	2.219 ± 0.142	34.533 ± 1.320	0.151 ± 0.002	2.380 ± 0.031	12.342 ± 0.501
**Early exponential**	0.206 ± 0.019	2.002 ± 0.137	37.799 ± 3.214	0.205 ± 0.011	2.320 ± 0.015 ****	28.919 ± 2.843 ****	0.149 ± 0.003	2.348 ± 0.080 ****	4.898 ± 1.092 ****
**Mid-exponential**	0.213 ± 0.008	2.038 ± 0.078	35.789 ± 3.588	0.165 ± 0.006	2.051 ± 0.053	25.307 ± 1.087 ****	ND	ND	ND
**Early stationary**	0.166 ± 0.126	1.956 ± 0.011	24.347 ± 2.719	0.184 ± 0.152	2.065 ± 0.028 *	26.528 ± 1.200	ND	ND	ND
**Stationary**	0.194 ± 0.172	2.038 ± 0.128	30.193 ± 2.661	0.183 ± 0.005	2.256 ± 0.012 **	17.217 ± 1.755 ****	ND	ND	ND
	**QA23**								
**Lag**	-	-	-	0.192 ± 0.007	2.252 ± 0.066	37.194 ± 0.759	0.177 ± 0.007	2.213 ± 0.007	10.960 ± 0.835
**Early exponential**	0.202 ± 0.004	2.153 ± 0.072	41.540 ± 0.858	0.195 ± 0.003	2.257 ± 0.011	36.560 ± 0.800 ****	0.175 ± 0.001 ****	2.055 ± 0.028	20.358 ± 0.610 ****
**Mid-exponential**	0.202 ± 0.002	2.252 ± 0.002	40.383 ± 0.566	0.196 ± 0.005	2.269 ± 0.006	33.896 ± 0.487 ****	0.162 ± 0.007 ****	2.256 ± 0.099	4.784 ± 0.114 ****
**Early stationary**	0.209 ± 0.001	2.327 ± 0.009	34.767 ± 0.937	0.188 ± 0.005 ****	2.286 ± 0.029	24.019 ± 0.917 ****	0.158 ± 0.003 ****	2.389 ± 0.017	16.695± 0.151 ****
**Stationary**	0.192 ± 0.005	2.301 ± 0.073	30.262 ± 0.651	0.184 ± 0.004	2.269 ± 0.015	16.985 ± 0.373 ****	ND	ND	ND

Table S3. Effect of different ethanol concentrations (0%, 8%, 10%, 12%) on mortality and ROS (reactive oxygen species) accumulation over time in the BY4743 and QA23 strains. Mortality rate expressed as the percentage of dead cells, ROS accumulation expressed as the geometric mean (Gmean). Mean and standard deviations are expressed. * indicates significant differences between stressed and nonstressed conditions (* for p-value < 0.05; ** for p-value < 0.01, *** for p-value < 0.001 and **** for p-value < 0.0001). ND stands for not determined.

**Ethanol concentration (%)**	**Control**	**8% ethanol**	**10% ethanol**	**12% ethanol**
		**MORTALITY**		
Time (h)	**BY4743**			
2	0.185% ± 0.001	2.300% ± 0.003 ***	1.293% ± 0.001	11.684% ± 0.008 ****
5	0.000% ± 0.001	2.570% ± 0.002 ****	2.331% ± 0.005 ***	22.610% ± 0.008 ****
20	2.033% ± 0.003	1.980% ± 0.002	38.858% ± 0.008 ****	57.760% ± 0.012 ****
	**QA23**			
2	0.040% ± 0.000	0.928% ± 0.001	1.908% ± 0.003 ***	2.477% ± 0.005 ****
5	0.031% ± 0.000	1.893% ± 0.006 ***	8.237% ± 0.001 ****	4.698% ± 0.005 ****
20	0.122% ± 0.001	1.488% ± 0.005 **	11.693% ± 0.006 ****	45.452% ± 0.003 ****
		**ROS**		
	**BY4743**			
2	78.52 ± 4.766	187.663 ± 10.573 ****	662.523 ± 17.919 ****	ND
5	96.18 ± 4.411	305.968 ± 13.779 ****	815.820 ± 9.322 ****	2090.846 ± 14.479****
15	267.925 ± 9.044	281.0410 ± 15.871	780.4388 ± 18.813****	714.902 ± 9.811 ****
20	296.800 ± 5.896	202.378 ± 8.953 ****	514.763 ± 2.710 ****	523.413 ± 12.363 ****
24	ND	212.163 ± 17.824 ****	618.824 ± 1.404 ****	601.414 ± 15.962 ****
48	350.993 ± 40.646	257.092 ± 3.6991 ****	310.107 ± 4.366 *	383.544 ± 23.821
	**QA23**			
2	85.417 ± 7.511	395.13 ± 32.075 ****	313.927 ± 46.076 ****	547.260 ± 0.042 ****
5	75.287 ± 7.252	418.49 ± 36.189 ****	394.243 ± 66.910 ****	599.470 ± 23.787 ****
8	111.330 ± 7.366	ND	453.745 ± 20.541 ****	579.390 ± 72.549 ****
20	136.637 ± 5.452	77.213 ± 6.0588	625.407 ± 30.025 ****	458.715 ± 21.376 ****
48	127.033 ± 8.621	113.74 ± 0.3182	148.470 ± 26.092	140.045 ± 3.401

Table S4. Effect of 8% ethanol on the *S. cerevisiae* strains BY4743 and QA23 on lipid peroxidation (nmol TBARS/mg protein), catalase activity ((H_2O_2 consumed/10 min)/mg protein) and superoxide dismutase (SOD) activity (U/mg protein). The parameters were calculated at different growth phases after the stress exposure: lag, early exponential (EE), mid-exponential (ME), early stationary (ES) and stationary (S). Lag phase was not observed for nonstressed cells. Mean and standard deviations are expressed. * indicates significant differences between stressed and nonstressed conditions (* for *p*-value < 0.05; ** for *p*-value < 0.01, *** for *p*-value < 0.001 and **** for *p*-value < 0.0001). TBARS stands for thiobarbituric acid-reacting substances.

	**BY4743**		**QA23**	
**Phase/condition**	**Control**	**8% ethanol**	**Control**	**8% ethanol**
		**TBARS (nmol TBARS/mg protein)**		
**Lag**		0.349 ± 0.000		0.491 ± 0.021
**EE**	0.218 ± 0.022	0.445 ± 0.141	0.348 ± 0.024	0.295 ± 0.009 *
**ME**	0.365 ± 0.033	0.438 ± 0.017 *	0.370 ± 0.000	0.621 ± 0.087 **
**ES**	1.011 ± 0.007	0.418 ± 0.012 ****	1.005 ± 0.088	0.508 ± 0.011 ***
**S**	1.031 ± 0.018	0.434 ± 0.045 ****	0.292 ± 0.024	1.664 ± 0.170 ***
		**CATALASE ACTIVITY ((H_2O_2 consumed/10 min)mg protein**		
**Lag**		0.704 ± 0.063		7.284 ± 0.282
**EE**	0.077 ± 0.022	1.843 ± 0.047 ****	2.777 ± 0.081	4.268 ± 0.192 ***
**ME**	-0.095 ± 0.002	7.117 ± 0.781 ****	1.417 ± 0.120	3.324 ± 0.792 *
**ES**	2.934 ± 0.350	74.006 ± 2.306 ****	62.700 ± 6.974	44.984 ± 12.396
**S**	21.888 ± 0.611	112.239 ± 2.929 ****	114.309 ± 1.881	114.550 ± 7.779
		**SOD ACTIVITY (U/mg protein)**		
**Lag**		23.839 ± 2.059		23.748 ± 1.800
**EE**	19.551 ± 0.933	27.712 ± 1.243 ***	16.990 ± 1.459	22.229 ± 0.492 **
**ME**	25.206 ± 0.483	27.560 ± 1.170 *	16.761 ± 1.301	26.142 ± 0.623 ***
**ES**	29.190 ± 1.469	43.739 ± 0.638 ****	28.758 ± 2.340	25.518 ± 1.590
**S**	34.743 ± 2.710	39.070 ± 0.260	15.429 ± 1.264	43.740 ± 3.279 ***

Table S5. Effect of melatonin supplementation (0, 5, 25, 50 μM) on the growth curve and recovery of *S. cerevisiae* strains BY4743 and QA23 after exposure to 8 % ethanol. The parameters analysed were growth rate (h^{-1}), maximum OD (ODmax) and area under the curve (AUC) until 20 h (growth curve) and 12 h (recovery) of growth. These parameters were calculated at different growth phases after the stress exposure: lag, early exponential (EE), and early stationary (ES). ND: Not determined. Mean and standard deviation (SD) values are represented, * indicates significant differences between stressed and nonstressed conditions (* for *p*-value < 0.05; ** for *p*-value < 0.005; *** for *p*-value < 0.001 and **** for *p*-value < 0.0001).

**Melatonin concentration (μM)**		**0**	**5**	**25**	**50**
			**GROWTH CURVE**		
**Parameter**			**BY4743**		
**rate**		0.065 ± 0.001	0.063 ± 0.001	0.073 ± 0.002***	0.069 ± 0.001*
**OD max**		2.164 ± 0.001	2.170 ± 0.005	2.174 ± 0.006	2.182 ± 0.004**
**AUC**		44.170 ± 1.637	41.550 ± 1.605	49.546 ± 2.620*	46.319 ± 0.148
		**QA23**			
**rate**		0.083 ± 0.006	0.083 ± 0.003	0.089 ± 0.001	0.081 ± 0.005
**OD max**		1.884 ± 0.013	1.529 ± 0.068***	1.938 ± 0.007	2.245 ± 0.092***
**AUC**		73.621 ± 2.156	63.569 ± 1.024***	80.771 ± 1.480**	80.669 ± 2.595**
			**RECOVERY**		
			**BY4743**		
**rate (h^{-1})**	**Lag**	0.202 ± 0.003	0.209 ± 0.008*	0.196 ± 0.008	0.193 ± 0.005
	**EE**	0.205 ± 0.011	0.221 ± 0.013	0.207 ± 0.017	0.225 ± 0.016
	**ES**	0.184 ± 0.002	0.188 ± 0.003	0.182 ± 0.003	0.173 ± 0.003
**OD_{max}**	**Lag**	2.219 ± 0.111	2.326 ± 0.031	2.284 ± 0.026	2.272 ± 0.028
	**EE**	2.320 ± 0.015	2.336 ± 0.034	2.333 ± 0.064	2.292 ± 0.004
	**ES**	2.065 ± 0.006	2.119 ± 0.002	2.098 ± 0.018	1.986 ± 0.001
**AUC**	**Lag**	34.533 ± 3.305	34.596 ± 1.409	31.787 ± 1.474	29.415 ± 0.391*
	**EE**	28.919 ± 2.457	38.326 ± 3.436****	40.700 ± 1.770*	45.775 ± 2.355****
	**ES**	26.528 ± 0.532	31.449 ± 0.460*	27.928 ± 1.520	27.717 ± 0.681

(continues in next page)

		QA23			
**rate (h^{-1})**	**Lag**	0.192 ± 0.007	0.202 ± 0.006	ND	0.201 ± 0.010
	**EE**	0.195 ± 0.003	0.198 ± 0.005	0.196 ± 0.003	0.193 ± 0.004
	**ES**	0.188 ± 0.003	0.187 ± 0.003	0.189 ± 0.013	0.187 ± 0.008
**OD max**	**Lag**	2.252 ± 0.066	2.302 ± 0.060	ND	2.242 ± 0.015
	**EE**	2.257 ± 0.011	2.288 ± 0.035	2.430 ± 0.178 *	2.331 ± 0.078
	**ES**	2.286 ± 0.015	2.315 ± 0.013	2.417 ± 0.088	2.301 ± 0.010
**AUC**	**Lag**	37.211 ± 0.729	39.834 ± 0.682	ND	34.414 ± 0.558
	**EE**	36.560 ± 0.800	39.611 ± 0.770	40.169 ± 3.120 *	38.547 ± 2.635
	ES	24.019 ± 0.479	22.667 ± 1.637	27.785 ± 1.725 *	23.654 ± 0.604

Table S6. Effect of melatonin (Mel) supplementation (5 or 50 μM) on BY4743 and QA23 cells exposed to 8% ethanol until lag, early exponential (EE), mid-exponential (ME), early stationary (ES) and stationary (S) phases, on mortality, ROS (reactive oxygen species) accumulation, lipid peroxidation, catalase and superoxide dismutase (SOD) activity. The parameters are expressed as ratio of the values of stressed cells with Mel vs stressed cells without Mel. Lag phase was not observed for nonstressed cells. Mean and standard deviations are expressed. * indicates significant differences with respect to stressed cells without melatonin (* for p-value < 0.05; ** for p-value < 0.01, *** for p-value < 0.001 and **** for p-value < 0.0001). TBARS stands for thiobarbituric acid-reacting substances.

	**BY4743**		**QA23**	
**Phase/Condition**	**5 Mel 8% ethanol**	**50 Mel 8% ethanol**	**5 Mel 8% ethanol**	**50 Mel 8% ethanol**
		**MORTALITY**		
Lag	-0.370 ± -0.019*	-0.402 ± -0.071*	-0.714 ± -0.345*	-0.771 ± -0.413*
EE	-0.371 ± 0.000*	-0.402 ± -0.036**	-0.702 ± -0.360**	-0.702 ± -0.225*
ES	-0.004 ± -0.001	-0.493 ± -0.058*	-0.369 ± -0.176	-0.542 ± -0.187
		**ROS**		
Lag	-0.052 ± 0.028	-0.114 ± 0.037	0.006 ± 0.052	0.069 ± 0.120
EE	0.032 ± 0.167	0.072 ± 0.368	-0.222 ± 0.040	0.034 ± 0.044
ME	-0.212 ± 0.096	-0.264 ± 0.043	-0.355 ± 0.122**	-0.604 ± 0.098**
ES	-0.310 ± 0.008**	-0.163 ± 0.007*	-0.328 ± 0.060**	-0.168 ± 0.069
S	-0.114 ± 0.043	0.027 ± 0.059	-0.149 ± 0.152	-0.288 ± 0.072*
		**TBARS**		
Lag	-0.124 ± 0.003**	-0.162 ± 0.009***	-0.244 ± 0.029*	-0.079 ± 0.008
EE	-0.056 ± 0.007	-0.062 ± 0.026	-0.173 ± 0.029	-0.093 ± 0.010
ES	-0.219 ± 0.014*	-0.043 ± 0.004	-0.122 ± 0.017	0.008 ± 0.000
		**CATALASE ACTIVITY**		
Lag	-0.078 ± 0.004	-0.273 ± 0.012****	-0.066 ± 0.005	0.014 ± 0.001
EE	-0.105 ± 0.009	0.373 ± 0.010***	0.087 ± 0.005	-0.025 ± 0.001
ES	-0.140 ± 0.003****	0.030 ± 0.000	0.179 ± 0.003	0.072 ± 0.009
		**SOD ACTIVITY**		
Lag	0.017 ± 0.000	0.004 ± 0.000	-0.244 ± 0.017*	-0.064 ± 0.006
EE	-0.094 ± 0.003*	-0.034 ± 0.002	0.084 ± 0.001*	-0.090 ± 0.001*
ES	-0.081 ± 0.001*	-0.103 ± 0.002**	0.001 ± 0.000	0.202 ± 0.023
